# Energy-Efficient and Robust QoS Control for Wireless Sensor Networks Using the Extended Gur Game †

**DOI:** 10.3390/s25030730

**Published:** 2025-01-25

**Authors:** Xiaoyang Zhong, Yao Liang, Yimei Li

**Affiliations:** Department of Computer Science, Luddy School of Informatics, Computing, and Engineering, Indiana University, 535 W. Michigan Street, IT 400, Indianapolis, IN 46202, USA

**Keywords:** wireless sensor networks, network control, quality of service, enhanced Gur game, energy efficiency, robustness

## Abstract

Outdoor wireless sensor networks (WSNs) operate autonomously in dynamic and unattended real-world environments, where sensor nodes are typically powered by their batteries. In hash outdoor settings, such as mountainous regions or underwater locations, recharging or replacing sensor node batteries is particularly challenging. For these WSN deployments, ensuring quality of service (QoS) control while conserving energy is crucial. This paper presents a novel QoS control algorithm for WSNs, built on extensions to the Gur game framework. The proposed approach not only enhances QoS performance compared to existing Gur game-based WSN control algorithms but also addresses their fundamental energy consumption challenges, enabling sustainable communication and extended network lifetimes. We evaluate the approach through comprehensive TinyOS-based WSN simulations and comparisons with existing algorithms. The results demonstrate that our approach, referred to as the robust Gur game, significantly enhances QoS control and achieves a 27.33% improvement in energy efficiency over the original Gur game and shuffle algorithms, showcasing the significant benefits of the proposed method.

## 1. Introduction

Wireless sensor networks (WSNs) have being widely deployed for various real-world applications, including environmental monitoring, event detection, and target tracking (e.g., [1,2,3,4]). To operate autonomously in dynamic and unattended real-world environments in those applications, it is essential that WSNs can support desirable quality of service (QoS) through some sophisticated control mechanisms, enabling WSNs to adapt to various factors such as tasks’ changing requirements, network conditions, node failure, and node mobility in real-time.

Typically, redundant WSN deployment is adopted to achieve the required QoS for extended network lifetime. The basic idea is that having redundant sensor nodes covering the same region makes the network system resilient to node failures, and network and/or environmental changes. Due to the redundancy in WSN deployment, only a portion of the deployed sensor nodes are needed to actively generate observation data/reports and transmit them to the sink, whereas the other sensor nodes can be idle to conserve their energy for sustainable and green communication. In this paradigm, QoS of the WSN is achieved by keeping a sufficient number of sensor nodes active in the network to conduct sensing and reporting at any time. The sink needs to receive a sufficient number of reports that present reliable information for its decision making. If the number of active sensor nodes is too few, the uncertainty increases in the decision-making process of the sink due to the lack of sufficient information collected by the WSN. On the other hand, if the number of active sensor nodes is too many, it would not only waste nodes’ energy, but may also cause potential traffic congestion in the WSN, resulting in fewer reports being received at the sink and, hence, the decrease in QoS.

Thus, the fundamental question to address in the QoS support of WSN is how to effectively control the number of active WSN nodes that perform sensing and transmitting data/reports to the sink under dynamic network conditions and environments. Clearly, it would be desirable that the devised solution should be distributed and autonomous, where all sensor nodes and the sink can simply work cooperatively in a self-organizing manner. For example, due to node failures and mobility, it is highly likely that the WSN sink has no accurate information about the total current number of available sensor nodes, except for the predefined optimum total number of reports needed in each decision-making period and the initial total number of deployed nodes. Similarly, individual sensor nodes may not know the optimum number of reports needed by the sink for the application, and also may not be aware of each other either.

### 1.1. Related Works

Iyer and Kleinrock [5] first proposed a QoS control for sensor networks based on the so-called Gur game to produce self-optimization [6,7]. The Gur game typically works on one-hop WSNs with star-topology [5], in which each sensor node can communicate with the sink. When the scale of a network is large, usually clusters can be effectively organized so that the Gur game can be applicable to each cluster. Liu et al. introduce Gur game framework to participatory sensing using smart phones [8]. A survey of game theory for WSNs can be found in [9]. However, the original Gur game approach is not power-efficient, and once the WSN reaches the optimum number of active nodes in the system, those active sensor nodes will remain active for every subsequent epoch until their battery energy is depleted or the optimum number of active nodes is changed by the sink. Besides, the original Gur game also suffers some convergence problems [10]. Previous works attempted to address the power balance and consumption issue of the original Gur game approach include an ACK strategy [11,12,13], the Gureen game [14], the shuffle algorithm [15], and the sensor activity control algorithm (SACA) [16]. Other recent works focused on the applications of Gur game-based frameworks to other problems, including finding maximum clique in social networks [17], and device management for recharging [18].

### 1.2. Our Work

In this study, we investigate a novel QoS control algorithm for WSNs, based on our extended and modified Gur game. This extension addresses key limitations of the original Gur game, introducing innovative mechanisms such as sensor node rotation, a proactive referee, and unambiguous reward/punishment strategies. These enhancements improve energy efficiency, resolve the convergence issues, and enhance the reliability of the Gur game-based QoS control.

Unlike prior works (e.g., [5,7,11,12,13,14,15,16,17,18]), which primarily rely on numerical analysis or high-level algorithmic simulations, our approach is thoroughly evaluated through realistic, low-level network emulations/simulations that capture intricate communication dynamics of WSNs in this study. Our results demonstrate that the extended Gur game significantly outperforms both the original Gur game and the shuffle algorithm [15] in terms of QoS and energy efficiency.

This paper significantly extends our previous conference publication [14], providing substantial new contributions, including theoretical insights, real WSN platform implementation, and extensive performance evaluations. The key contributions of this work are summarized as follows:

- Innovative QoS control mechanisms: We propose an extended Gur game, featuring sensor node rotation, a proactive referee, and unambiguous reward/punishment mechanisms, specifically designed for energy-efficient and robust QoS control in WSNs.
- Theoretical advancement: We prove that the proposed extension resolves the M/2 convergence issue, an open problem in the original Gur game model.
- Real-world implementation: Unlike earlier studies confined to algorithm-level discussions, we implement the extended Gur game on a real WSN platform using TinyOS and nesC, validating its practicality and effectiveness through real-world deployment.
- Enhanced performance evaluations: Extensive evaluations using realistic network emulations and system-level simulations provide high-fidelity performance insights, demonstrating significant improvements in QoS and energy efficiency.
- Robustness under node mobility: Additional evaluations under dynamic node mobility highlight our approach’s robustness and its applicability in challenging, real-world scenarios.

The remainder of the paper is organized as follows. Section 2 provides some background of the Gur game. Section 3 presents our extended Gur game approach for energy-efficient and robust QoS control for WSNs. Section 4 describes the simulation study with QoS performance results and analysis, while Section 5 provides the simulation evaluation on energy efficiency. Finally, our conclusions and future work are given in Section 6.

## 2. Background

**Figure 1:**
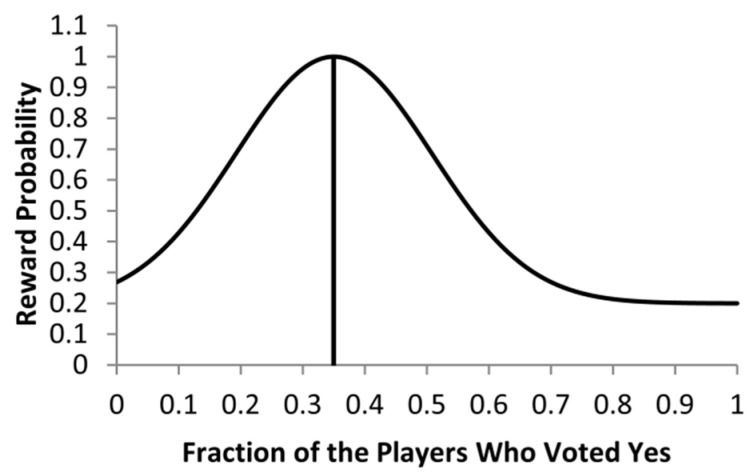
A typical reward function used in the Gur game, where the total number of players is 100, and the desirable fraction is 0.35.

The Gur game is a mathematical model introduced in [6] for self-control in a cooperative environment. To understand the principle of the Gur game algorithm, let us consider a simple example [5,7]. Assume that we have M players and a referee. The players are independent and are not aware of each other. In every round, the referee asks the players to vote yes or no, and counts up the yes and no answers. The referee then calculates a reward probability P=p(f) as a function of f, the ratio of the players who voted yes in the last round to the total M players in the designed game size. The reward probability P will be broadcasted to the players before the starting of the next round. Each player is independently rewarded (with the probability of P) or penalized (with the probability of 1-P) regardless of how it voted in the last round. A typical reward probability function is shown in Figure 1. The maximum of reward probability function occurs at the optimal ratio (i.e., $f^{*}$) to the total M players, which corresponds to the optimal number of players who voted yes in the previous round. It can be shown that a finite state automaton can be constructed with each individual player such that there will be overall about $f^{*}$ of M players to vote yes after sufficient rounds in the game. We say that the Gur game “converges” to the optimal state if exactly $f^{*}$ of the M players vote yes.

**Figure 2:**
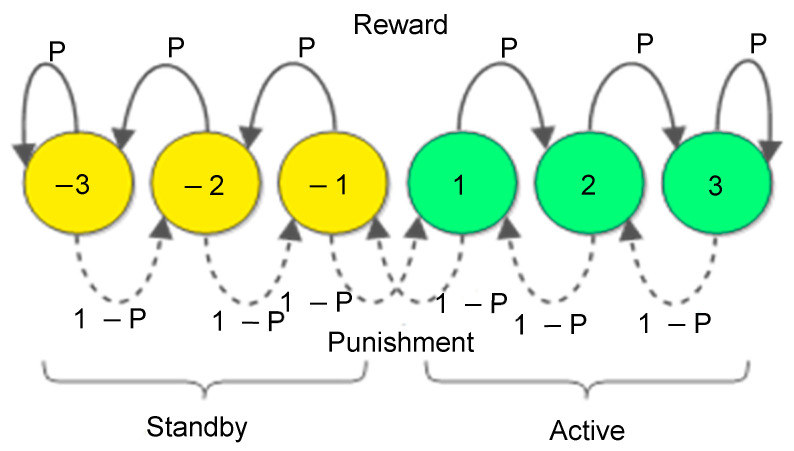
A finite state automaton associated with each sensor node in the Gur game control strategy. A sensor node’s automaton can be constructed with 2N states (here N=3).

When the Gur game is applied to WSN for QoS control, each sensor node acts as a player, and the sink acts as the referee. In many WSN applications, a certain number of sensor nodes periodically and independently report their samples to the WSN sink. A sensor node can be either active or standby. One can construct a finite state automaton of 2N states by assigning N active states and N standby states to each sensor node, as shown in Figure 2, where N = 3, with positive number and negative number indicating the active and the standby states, respectively. When a node is active, it sends reports (i.e., voting yes) to the sink at each round, whereas a standby node does not send any report (i.e., voting no). The sink then counts the number of reports received in the last round as yes votes, and calculates and broadcasts the reward probability to all sensor nodes for the next round. To ensure the reliability and efficient energy conservation for the sensor nodes, the number of independent reports received by the sink should fall within a small interval around an optimal value—neither significantly fewer nor significantly more. Upon reception of the broadcasted reward probability, sensor nodes transit their states accordingly, sending or not sending. This process of network control is repeated for each round within the network.

A sensor node transits its state according to the reward probability spread by the sink. As shown in Figure 2, a sensor node transits its state along the solid arrowed line with the reward probability of P, or transits its state along the dotted arrowed line with the probability of 1-P. Thus, a reward pushes a node moving toward the “edge” states while a punishment pushes a node moving toward the “center” states to switch between its active and standby states. The Gur game principle guarantees that nodes will pin to the edge states after sufficient rounds.

There are several important observations about the convergence of Gur game [10]. First, the Gur game in general converges to the vicinity of the optimal state. That is, the convergence ratio $f^{c}$ satisfies the inequality $\left|f^{c}-f^{*}\right|\leq \varepsilon$, where ε>0 is a constant. Second, when N=1, that is, when each player only has two states, the Gur game in general cannot converge. Instead, it will fluctuate around the ratio of 1/2 rather than the optimal ratio $f^{*}$, which is called the M/2 problem. Third, the game lingers near the $f^{*}$ longer as N increases, since an automaton must march back through a longer chain before reaching −1 or +1 to change its votes. In a realistic wireless environment, the optimal state could easily be disturbed by many factors. For instance, packet losses and duplicates may cause inaccurate counting of the number of active players and results in an inaccurate reward probability, which may pull the Gur game away from the optimal state.

Now assume a sensor network initially with n nodes is randomly deployed over the target monitoring area, and a sink/base station. A unique feature of Gur game-based WSN control is that the sink only needs to be aware of the optimal number of nodes (i.e., the optimal ratio $f^{*}$ of the designed network size) to be needed to maintain the desired QoS level, even though it does not know currently the exact total number of available nodes in the network due to nodes’ mobility and failure. A typical reward function in the Gur game is given as follows:

(1) $$P=p\left(f\right)=0.2+0.8e^{-20{\left(f-f^{*}\right)}^{2}}$$

where f is the ratio of nodes that voted yes in the previous round to the designed network size. Assume $f^{*}=0.35$, the reward function is shown in Figure 1. The Gur game starts by initially setting every node’s state to be standby (−1). For instance, in the beginning, the sink calculates the reward probability p(0)=0.27, and then broadcasts this reward probability value in the network. After receiving it, each node generates a random number that uniformly distributed in range [0, 1] independently. If the random number generated by a node is smaller or equal to the received reward probability, it will reward itself and move toward the edge; otherwise it will punish itself by making one state transition toward the center, or switching its current state to the opposite state when either at −1 (standby) state or +1 (active) state. Nodes at active states will make observations (e.g., samplings) and send their reports to the sink. In turn, the sink will recompute the reward probability accordingly and start the next round, and the game goes on. After sufficient rounds, the Gur game converges to the vicinity of the optimal state, in which nearly $f^{*}$ of the M nodes will be at their active states, the other nodes being at their standby states. The nodes can hardly change their states in the subsequent rounds because the reward probability is nearly $p\left(f^{*}\right)=1$. Thus, all nodes will stay at either active or standby until a significant change occurrs on the number of active nodes, which may be caused by nodes’ energy depletion or nodes’ mobility, breaking the stability of the system.

## 3. The Extended Gur Game

In the WSN QoS control based on standard Gur game, nodes in standby states need to be awake for idle listening and receiving, although they do not transmit. Idle listening, receiving, and transmitting consume nearly the same amount of energy [19]. In fact, a node spends most of the time and energy for idle listening. Our approach to improve a node’s energy efficiency is to put the node into deep sleep state to eliminate any unnecessary idle listening. In the standard Gur game, once the QoS of a system reaches its optimal level, none of the sensor nodes change their active or standby state until some of the active nodes are running out of their batteries or moving away. During this process, all the standby nodes must stay idle listening in order to receive the reward probability broadcasted by the sink. Therefore, almost all the standby nodes’ energy is wasted, which is the main problem of the standard Gur game. Besides, the standard Gur game also suffers some reliability weakness, which will be illustrated later in the paper. To address these issues, we propose an extended Gur game, referred to as robust Gur game, for energy-efficient and robust QoS control in WSNs. Our approach introduces three novel concepts: player rotation, a proactive referee, and unambiguous reward/punishment mechanisms. We demonstrate that the robust Gur game significantly enhances the reliability of QoS performance compared to the standard Gur game. Furthermore, we show that our robust Gur game is far more energy-efficient than the standard Gur game and the modified Gur game shuffle, enabling sensor nodes to conserve energy and substantially extending the WSN’s lifetime.

In the following sections, we present our robust Gur game model. Section 3.1 introduces player rotation within the game, followed by the robust Gur game proactive referee in Section 3.2. Section 3.3 outlines our approach to unambiguous reward/punishment. Finally, in Section 3.4, we formulate the extended Gur game, integrating the three introduced concepts.

### 3.1. Player Rotation

We introduce sleep states into each finite state automaton that realizes the game. The idea is to allow players (e.g., sensor nodes in WSN) to have the ability to take a break from the game. Sleep brings some drastic changes to the game. When a player is in a sleep state, it does not participate in any game activities (e.g., idle listening, receiving, or state transition) and, thus, conserves its resources (e.g., energy). A timer is created and associated with a player’s current active or standby state. When the timer expires, the player goes to sleep and temporarily leaves the game. In order to bring the player back to the game, another timer is created to wake the player up for rejoining the game. Furthermore, to better balance the workload among the players, we consider that when a player was active before its rotating out to sleep, it will rotate into standby after its waking up, and vice versa.

### 3.2. A Proactive Referee

Because players periodically rotate out due to the player rotation mechanism introduced above, the extended Gur game will no longer keep its stable state after reward probability P reaches one as in the standard Gur game. Whenever the stability of game is broken, certain turbulent dynamics of the game would be triggered to move the system back into stability after some rounds. This rotation-triggered dynamics should be minimized as much as possible to maintain the QoS level of the system. Normally, the referee can only be reactive to the total yes votes in the game, which means the referee can only react after player(s) rotate out. To improve the performance of the game, we devise the proactive referee in our robust Gur game, based on players’ notification of their expected rotation (according to timer) in advance. This way, the referee can take predictive correction action by updating reward probability ahead, which helps to smooth the rotation-triggered dynamics out in the game. In contrast, the reactive referee would have to incur one-round extra delay to react upon any player’s rotation.

### 3.3. Unambiguous Reward/Punishment

**Figure 3:**
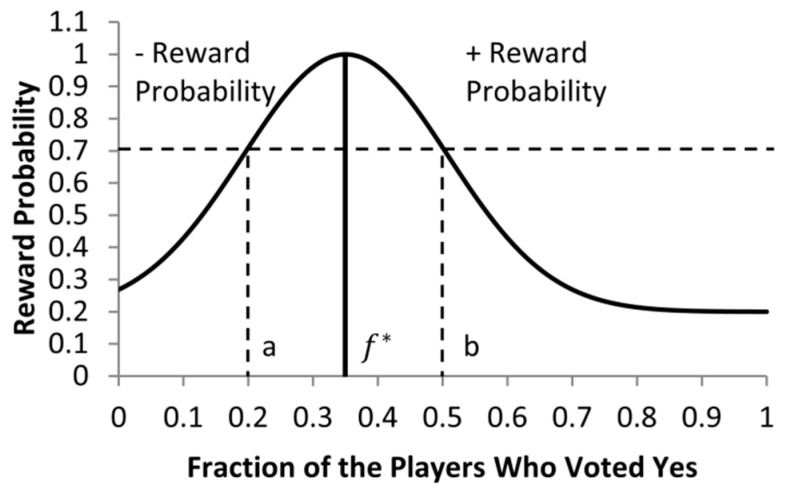
An illustration of two numbers of yes votes $a<f^{*}$ and $b>f^{*}$, which would generate identical reward probability.

The original Gur game is somewhat ambiguous to each player with regard to whether to reward or punish itself to benefit the game when the received probability P<1. This situation is illustrated in Figure 3. For example, consider two game situations, a vs. b, as shown in Figure 3, where a<0.35, b>0.35, $f^{*}=0.35$, and p(a)=p(b)=P=0.7. For the situation of a, players should be encouraged to make their transitions from standby states to active states, rather than the opposite transitions from active states to standby states. On the other hand, for the situation of b, players should be encouraged to make their transitions from active states to standby states rather than the transitions in the opposite direction. However, in the original Gur game, when each player receives probability P from the referee, it has no clue whether the situation of the game is a or b due to the lack of information, and, thus, makes its transition blindly.

To address this drawback, our idea is to let the referee send out one extra bit of information when P<1, along with the reward probability, to indicate whether it is the situation of a or b. With this one extra bit flag, each player can act more favorably in the direction of improving the system’s performance. This flag bit of information can symbolize the “sign” of the reward probability: any “negative” probability indicating the situation of a, while any “positive” probability indicating the situation of b. When players receive a “negative” probability, implying no sufficient number of nodes in the “active” state, thus, only those standby players make transitions (reward/punishment) according to the probability (i.e., the absolute value of the probability) as in the original Gur game, whereas all active players are rewarded with the probability of one instead. Similarly, when players receive a “positive” probability, only those active players make transitions (reward/punishment) according to the probability, whereas all standby players are rewarded with the probability of one instead. Indeed, this extension of Gur game has solved the M/2 convergence problem—the open problem of the original Gur game model.

**Lemma** **1.** *Let each player (i.e., automaton) in a game have only two states (i.e., N = 1). Assume that the referee’s reward probability P(f) is a convex function of f, where 0 ≤ f ≤ 1, 0 ≤ P(f) ≤ 1, and $P\left(f^{*}\right)=1$. Assume that each player makes its state transitions according to the above proposed unambiguous reward/punishment mechanism and f changes along with epoch T. Then $\underset{T\to \infty }{\lim}\left|f\left(T\right)-f^{*}\right|=0$ for any given $f^{*}$ in the game.*

**Proof.**  See the Appendix A. □

### 3.4. Robust Gur Game for QoS Control in WSNs

**Figure 4:**
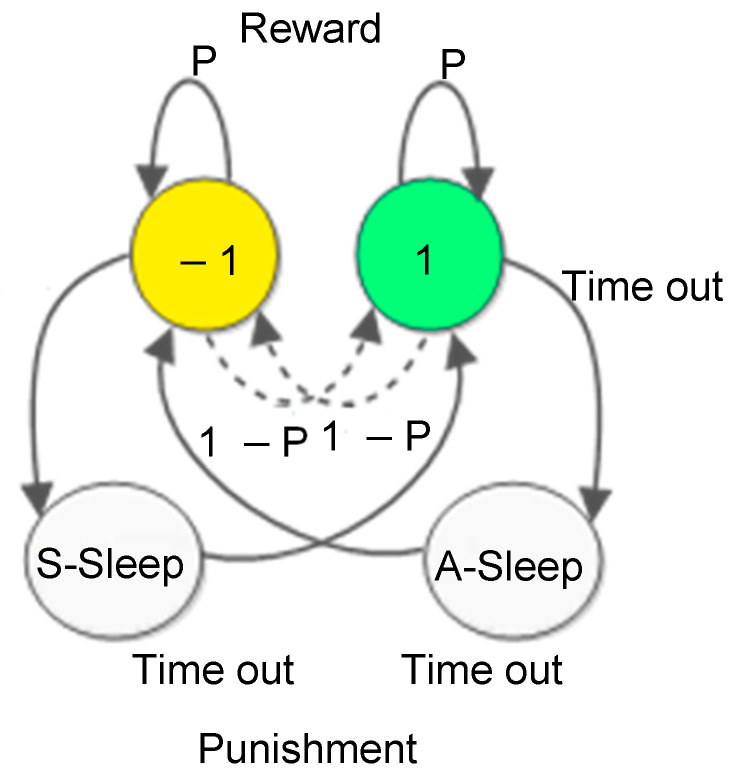
An improved finite state automaton associated with each sensor node in the robust Gur game control strategy.

The robust Gur game model for QoS control in WSNs is based on the three novel concepts and mechanisms introduced above. In the robust Gur game, each player (i.e., sensor node) is associated with a finite state automaton as shown in Figure 4. There are four states: active (+1), standby (−1), and two sleeps (S-sleep and A-sleep). Transitions between active state and standby states by being rewarded with probability P or punished with probability 1-P based on the reward probability sent by the sink (i.e., the referee). Every sensor node has a timer (with multiple configurable time-out parameters). A node in the active state moves to A-sleep state when the timer expires. A node in A-sleep will take a break (i.e., transceiver turned-off, microcontroller put in sleep) for certain cycles according to a time-out parameter and wakes up when the timer expires and moves to the standby state. Similarly, a node in the standby state moves to S-sleep state when its timer expires. A node in S-sleep will take a break for certain cycles according to a time-out parameter and wakes up when the timer expires and moves to active state. Thus, in general, there are four time-out parameters (i.e., active time-out, standby time-out, A-sleep time-out, and S-sleep time-out), which can be configured for each automaton.

## 4. Performance Evaluations

### 4.1. Development Environments and Simulation Tools

Unlike the previous works that primarily relied on numerical analysis and/or algorithm-level simulations, we have implemented the proposed energy-efficient and robust Gur game in TinyOS [20] using nesC language [21]. We conducted rigorous performance evaluations using network simulation tools that accounted for the full details of WSN communication protocols, the operating states of the radio transceiver (transmitting, receiving, idle listening, and sleep), and microcontroller instruction executions. This approach provides more accurate and reliable evaluation results, including a detailed energy efficiency analysis for green communication.

TinyOS [18] is an open-source operating system developed in the nesC language [21] and has been widely used in research community and real-world applications and testbeds (e.g., [22,23,24,25]). Unlike monolithic OSes, TinyOS is a set of reusable components that are included as-needed in applications to provide efficient and extremely low-power operations [20,26]. We implemented our robust Gur game and other Gur game algorithms with TinyOS version 2.1.2 for comparison. In this section, we evaluate the QoS performance of all the algorithms using the decent simulator TOSSIM [27] that provided by TinyOS, and study the energy efficiency using simulator Avrora [28] in the next section.

TOSSIM [27] is a mote simulator that compiles directly from TinyOS applications. An advanced feature of TOSSIM is its environmental noise modeling. TOSSIM takes an environmental noise trace as input and creates accurate noise model for each simulated sensor node. A commonly used real world environmental noise trace today is called as Meyer Heavy noise trace, which was taken at the Meyer library at Stanford during heavy 802.11 activities [29,30]. TOSSIM simulates network behavior (i.e., packet losses, duplicates) with high fidelity, however, it is unable to accurately simulate the per-instruction time and anything else related to it, including energy usage. Thus, we need to apply an additional simulator for accurate energy analysis.

Avrora [26] is a cycle-accurate instruction-level sensor network simulator that provides an accurate and realistic radio model of popular CC2420 transceiver [31] for IEEE 802.15.4 standard [32]. It emulates the sensor node’s hardware and runs the actual microcontroller programs. The binary file of a TinyOS program can be directly exported to an ELF file and be emulated using Avrora. State changes are tracked at cycle level, which provides the highest possible accuracy. Reference [19] shows that the simulated energy consumption in Avrora is approximately constantly 20% less than the actual energy consumption, independent of the radio state. This indicates that, with a straight forward calibration, Avrora can provide accurate and convincing energy estimation.

### 4.2. Simulation Setup

**Figure 5:**
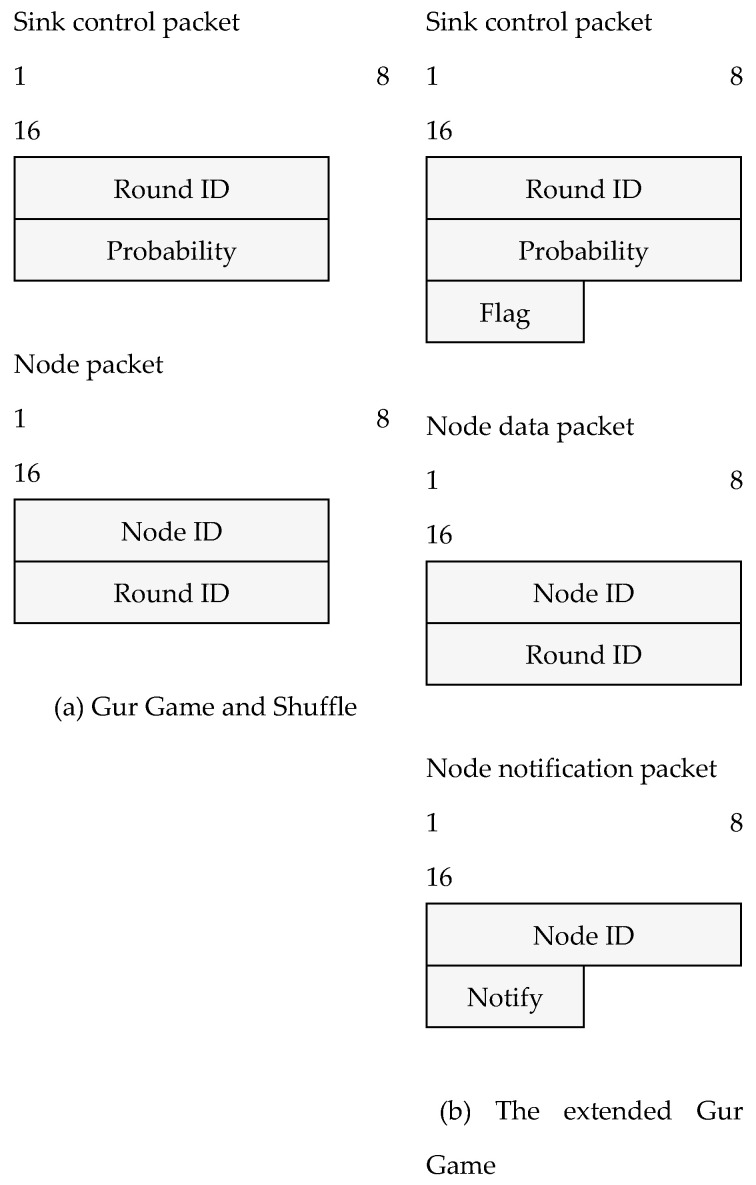
Packet structures of standard Gur game/shuffle) (**a**) and the extended Gur game (**b**).

In our simulations, we assume the network forms a star topology and all nodes send simple packets for communication. To ease the implementation, we designed several packet structures and operate on byte level instead of bit level. For example, the sink broadcasts the reward probability with an additional byte to indicate the unambiguous reward/punishment information even though a single bit is sufficient. The packet structures of the original Gur game/shuffle algorithm and our proposed robust Gur game are shown in Figure 5a and Figure 5b, respectively. Together with the CC2420 MAC layer header (which is 11 bytes) [32], the total packet size is less than 20 bytes.

The network had 100 sensor nodes with the optimal ratio $f^{*}=0.35$; a round interval (i.e., epoch) was set to 15 s. For TOSSIM simulation (QoS performance), each trial was 2000 rounds. The optimal ratio of 0.35 is a representative setup commonly adopted in previous research in the literature. In practice, this value depends on the level of redundancy required for the specific WSN deployment. A ratio of 0.35 (i.e., 35%) implies approximately two times redundancy in the deployment, which be considered reasonable for certain applications. The first 4000 lines of the Meyer Heavy noise trace were used in our simulation for creating noise model for each node, which introduces averagely 8% of packet losses. For Avrora simulation (energy efficiency), each trial was 10,000 s. Table 1 summarizes the simulation configurations.

### 4.3. Performance Metrics

To effectively evaluate the quality of control on the number of active sensor nodes in the network, we introduce a performance metric called QoS ratio, defined as follows:

(2) $$QoS=\frac{T_{QoS}}{T}$$

where *T* is the total number of rounds in the network; and *T_QoS_* is the total number of rounds in which the ratio f is maintained in a predefined satisfied level η of the optimal ratio $f^{*}$, i.e., $\left\lceil f^{*}\eta \right\rceil \leq f\leq \left\lfloor f^{*}(2-\eta )\right\rfloor$. For example, if $f^{*}=0.35$ for a given initial network size *M*, and η = 90%, then we have 0.315≤f≤0.385. That is, in any WSN round of decision making, the QoS level of the network is satisfied if the fraction of nodes that vote yes is controlled in the range of [0.315, 0.385] for the round. If the initial network size M is 100, the number of the active nodes must fall in range [32, 38] to satisfy the QoS requirement. The other performance metric we used for network QoS control is the average number of active nodes for a trial. In addition, we introduce the amount of energy consumption (simulated with Avrora) as the metric for energy efficiency.

### 4.4. The Evaluation of the Standard Gur Game

We mainly consider Gur game with N = 3 (i.e., three active states and three standby states) according to the previous work [5], which suggested that N = 3 would provide a good system performance. In addition, we also conducted simulations on the Gur game with N = 1, to better compare the original Gur game with our robust Gur game. Table 2 and Table 3 show the QoS performance results obtained from TOSSIM simulation for five trials of the Gur game with N = 1 and N = 3, respectively.

**Figure 6:**
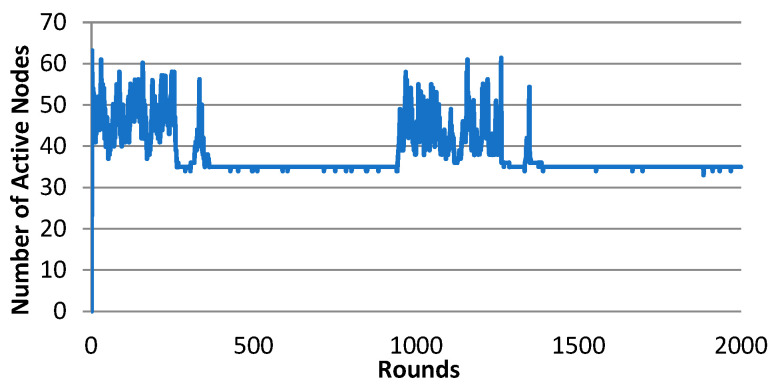
The number of active nodes of Gur game (N = 1), trial 2.

The Gur game with N = 1 behaves unstable because the M/2 problem occurs. This behavior is shown in Figure 6, where no trend of convergence was found until the system randomly hit the optimal state. After that, the system kept the QoS level since the reward probability was 1. For some other given optimal ratios, the Gur game with N = 1 may hardly hit the optimal state. For instance, if the optimal ratio is $f^{*}=0.25$, the system failed to hit the optimal state [14]. Even if it hits the optimal state, however, its QoS level is so fragile that a single packet loss or duplicate could possibly pull the system back to chaos. As illustrated in Figure 6, the system was pulled away from the QoS range at round 945 due to occasional packet losses, and had to spend hundreds of rounds to randomly hit the optimal state again.

**Figure 7:**
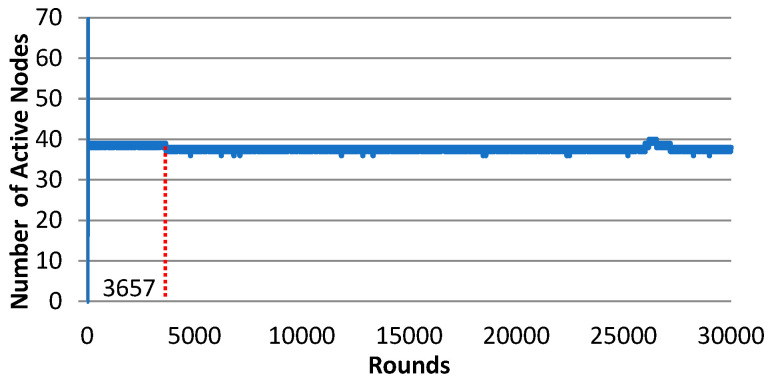
The number of active nodes of Gur game (N = 3), trial 5. The system converged to QoS range at round 3657.

With N = 3, for all trials the Gur game converges very fast with very good stability. The convergence position actually determines the system’s QoS performance. If the Gur game converges to the acceptable QoS range of [32, 38] (e.g., trials 1, 2, and 3), it achieves more than 99% QoS ratio. However, if it falls slightly out of the acceptable QoS range (e.g., trials 4 and 5), the QoS ratio then becomes very bad. To further examine the convergence behavior of Gur game with N = 3, we let the fifth trial continue for 30,000 rounds. As shown in Figure 7, at the beginning, the system quickly converged to 39 (i.e., 39 active nodes), and slowly approached to 38 at round 3657, which falls into the QoS range, and then stayed there until the end of the simulation. It is still unknown how long it would take in general for the Gur game with N = 3 to converge to the specified acceptable QoS range.

### 4.5. The Evaluation of Shuffle

Shuffle [15] swaps the states of the nodes at the edge states (i.e., +N, −N) in Gur game periodically in order to balance the energy consumption. The exchanging of states will destroy the system’s optimal state and, thus, create turbulence in the system. The time needed for the system to re-reach its optimal state depends on the value of N. The larger N, the longer the time. We conducted simulations according to the configuration from [15], where N = 2, and the time interval for swapping states is every 500 rounds.

**Figure 8:**
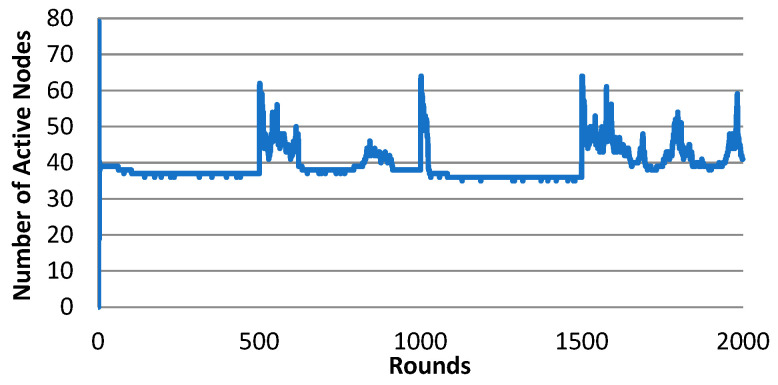
The number of active nodes of shuffle (N = 2), trial 2.

Table 4 shows the statistics of the QoS performance of shuffle for five trials. The average QoS ratio is 43.46% and the average number of active nodes is 40.35 for the five trials. The number of active nodes at each round for trial 2 is plotted in Figure 8. The system swapped the states three times during the simulation. After the first swapping (at round 500), the system took about 120 rounds to re-converge to the QoS range. After the second swapping (at round 1000), the system took about 25 rounds to re-converge to the QoS range. After the third swapping (at round 1500), the system failed to re-converge through the end of the simulation. This result indicates that the swapping has introduced too many disturbances to the system, so that the system, with a high probability, may not be able to re-converge during a limited time. With N = 3, shuffle would even have worse performance since it would take much longer time to re-converge.

### 4.6. The Evaluation of the Extended Gur Game

The proposed robust Gur game exploits the three novel extensions to enhance the performance of the original Gur game. While player rotation temporally breaks the game system’s optimal state to achieve better energy efficiency, the proactive referee and unambiguous reward/punishment help the system to converge quickly and maintain the QoS level.

Since the players rotate their states according to their respective timers, the time-out parameters play an important role for a successful game. We note that several principles should be satisfied to construct a proper time-out parameter configuration for the robust Gur game. First, the timer for the active state must be sufficiently long for the system to converge and behave optimal QoS performance. It is observed that the convergence time of the robust Gur game is usually within six rounds due to the unambiguous reward/punishment mechanism. Thus, we can set the length of the timer for the active state to multiples of the convergence time (in rounds). Second, when any nodes wake up from the S-sleep state and transit into the active state, the disturbance should be necessarily small so that the system can keep the QoS level. In this regard, the sleep timer should be randomized so that individual nodes would wake up separately to avoid a set of nodes entering the active state simultaneously. For instance, at time t, if there are 30 nodes in S-sleep state, and the time-out parameter is randomized over the range of [1, 60] rounds, then, on average, there is only 0.5 node to transit to the active state in every round, which has little influence on the system’s performance. A more theoretical analysis about the parameter selection is still an open question.

Table 5 lists some configurations of the robust Gur game, whose corresponding QoS results are given in Table 6. As we can see, configuration 2 produces the good QoS performance of the WSN, since it satisfies both configuration principles discussed above. Regarding energy efficiency, we calculated the average number of nodes that are awake in each single round. The result indicates that configuration 2 also seems to yield very good energy efficiency, since, on average, about 30% of the nodes are in deep sleep at each round. Hence, we select configuration 2, with both very good QoS performance and energy efficiency, for further simulation study. Table 7 shows the details of the simulation result with configuration 2.

### 4.7. Performance Comparison and Analysis

**Figure 9:**
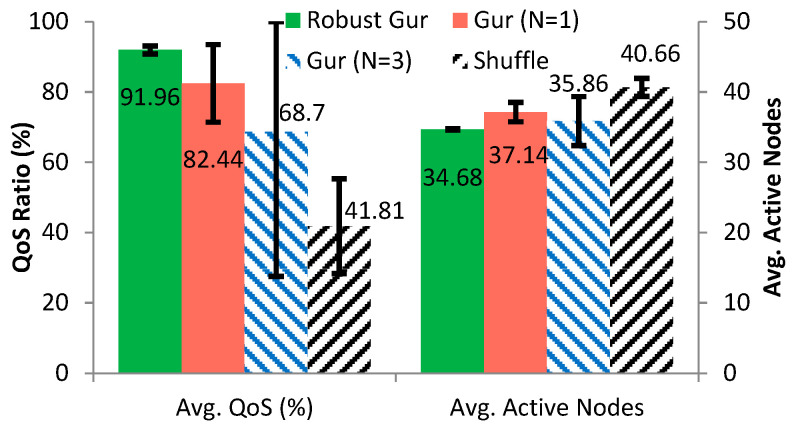
Comparison of different control schemes on the average QoS ratio (**left**), and average number of active sensor nodes (**right**), with corresponding standard deviation.

**Figure 10:**
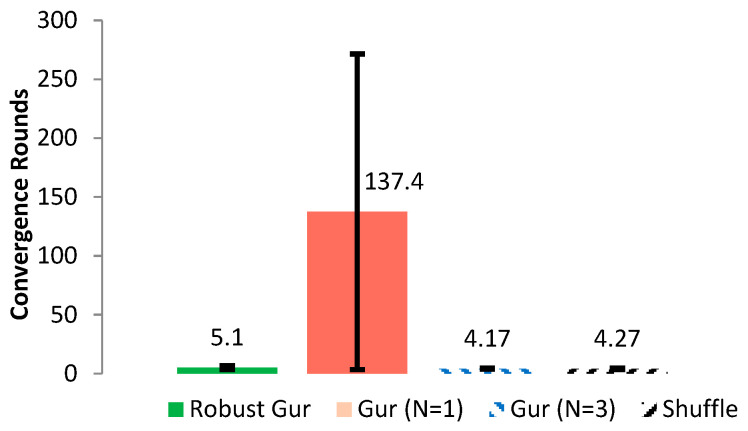
Comparison of different control schemes on the average number of rounds for the first convergence (as labelled) and the standard deviation.

To compare the QoS performance of the standard Gur game, shuffle, and our robust Gur game, we further conducted 30 simulations for each algorithm using TOSSIM. For the standard Gur game, configurations N = 1 and N = 3 were used in our comparisons. We chose N = 1 because the original Gur game encounters a convergence issue under this configuration, as noted in [10]. Our improved algorithm resolves this convergence problem, making it meaningful to evaluate and compare the performance of our algorithm with the original Gur game for N = 1. Additionally, prior research [11] has indicated that N = 3 often yields good overall system performance based on extensive research experience. As such, we selected N = 3 as a representative configuration to compare our algorithm against the known optimal configuration of the Gur game. The statistical results are shown in Figure 9 for the average QoS ratio and the average number of active nodes. It can be seen from Figure 9 that the robust Gur game has the best performance overall and, particularly, is very robust. It also demonstrates that the robust Gur game converges to a closer position to the optimal state than the Gur game (with either N = 1 or N = 3) and shuffle. The major problem of the Gur game with N = 3 is that it converges but not often into the given QoS range, which causes significant deviation on its performance, resulting in worse overall performance than the Gur game with N = 1. However, the Gur game with N = 1 is unstable and takes uncertain time to fall into the QoS range, as discussed in Section 4.4, which makes it undesirable in realistic environments. The convergence performance of each algorithm is illustrated in Figure 10, for the optimal ratio $f^{*}$ is set to 0.35. The Gur game with N = 1 usually takes hundreds of rounds for the first falling into the QoS range, whereas the other algorithms need only a few rounds.

**Figure 11:**
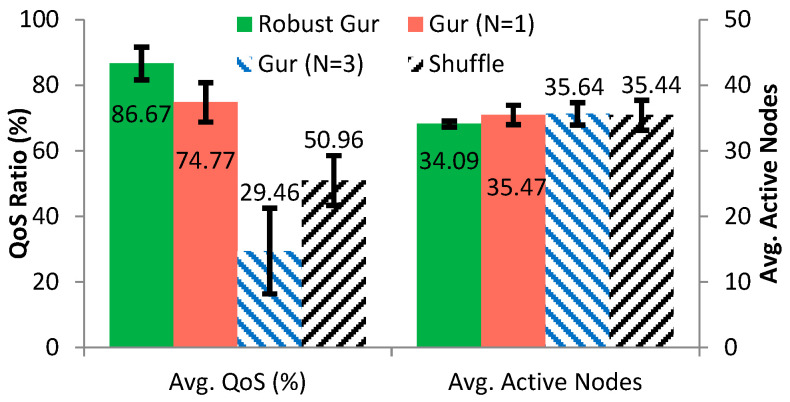
Performance comparison under node mobility for Gur game, shuffle, and robust Gur game.

To examine the robustness of Gur game, shuffle, and our robust Gur game under network mobility, we conducted simulations where nodes dynamically moved into and moved out the network. Initially, the network had 100 nodes plus one sink, among which 50 nodes were allowed to be mobile. Each simulation trial consisted of three phases. During the first phase, no nodes were mobile; in the second phase, 50 nodes moved out the network at random time points; in the third phase, all left nodes rejoined the network at random time points. Note that the second phase was overlapped in time with the third phase, so that both nodes leaving out and coming in simultaneously were simulated. In the simulation setup, when the number of mobile nodes left the network reached 35, the moved-out nodes began to come back. We conducted the simulation for 30 trials for each of the approaches, with 2000 rounds per trial. The simulation result is shown in Figure 11.

As we can see, the robust Gur game significantly outperforms the other approaches under network mobility. Furthermore, compared to its static scenario, the performance of robust Gur game only has a little decrease. In the robust Gur game, nodes always transit states to the direction that benefits the system due to its innovation of unambiguous reward/punishment mechanism. Thus, it is robust under the dynamics of network mobility. In contrast, the Gur game with N = 3 suffers the greatest drop in its the performance, due to the fact that most nodes of the system will be on the edge state of the automaton (i.e., either +3 or −3) when the system reaching the optimal operation range, thus making it difficult to switch their active or standby status promptly in response to nodes mobility in the system. On the other hand, although the performance of the Gur game with N = 1 did not decrease very significantly, it suffers the problem of convergence, as shown in Section 4.4, in which the system would be under drastic fluctuation.

## 5. Energy Efficiency

We conducted simulations using Avrora to examine the energy efficiency of each algorithm. For all the examined algorithms, the simulated network size was 100 (not including the sink) with optimal ratio $f^{*}=0.35$. The round interval was 15 s. Each trial was 10,000 s.

**Figure 12:**
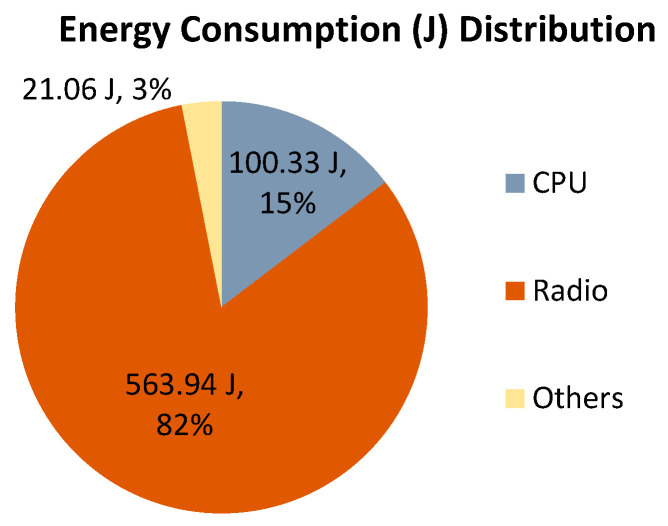
Energy consumption distribution of an idle listening node (the Others including the energy consumption of the default sensor board).

To begin with, a benchmark test was conducted in which a single node was idle listening for 10,000 s. Figure 12 shows the distribution of the energy consumption of the benchmark case; the total energy consumption was 685.33 Joule and 82% of the energy was consumed by idle listening. For CC2420 transceiver, the energy consumption of node’s receiving, transmitting, and idle listening is almost the same. Avrora does not distinguish receiving mode and idle listening mode.

Now consider the original Gur game. Since nodes never sleep in the original Gur game, no matter what state they might be in (standby or active), they consume nearly the same amount of energy as the benchmark, as shown by our simulation statistics listed in Table 8.

For a single node, most of the energy is consumed by the radio, and particularly, by receiving/idle listening. The details of the energy consumption of the most active node and the least active node for Gur game (N = 3) are shown in Table 9. Both nodes share a similar energy consumption distribution with the benchmark, as the transmitting energy consumption is negligible compared to the receiving/idle listening energy consumption.

Regarding the energy consumption of shuffle, similar to the original Gur game, since shuffle does not have any sleep states, its energy consumption is similar to that in the original Gur game and the benchmark.

For the energy consumption of robust Gur game, multiple simulations using Avrora were conducted, as shown in Table 10. The average number of awake nodes (i.e., active nodes and standby nodes together) is only 70.66 for the 100-node network. We observed on average 27.26% improvement compared to the benchmark. Note that when the node is in a sleep state, both the radio and the microcontroller are turned off. Since the sleep periods are randomized, individual node’s energy consumption varies from one another. However, they share a similar energy consumption distribution shown in Figure 12: most of the energy is consumed by the radio’s receiving/idle listening.

**Figure 13:**
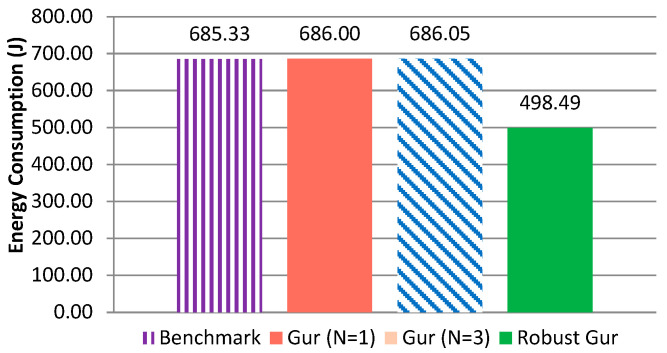
Comparison of different control schemes on average energy consumption (J).

Figure 13 shows the per node average energy consumption of the original Gur game, robust Gur game, and the benchmark (labeled as idle listening in Figure 13). The Gur game (either N = 1 or N = 3) consumes nearly the same amount of energy as the benchmark, since the nodes do not sleep. On the other hand, the robust Gur game is 27.33% more energy efficient than the original Gur game and shuffle. Moreover, Table 10 indicates that robust Gur game has on average 29.34% fewer awake nodes than that of the Gur game on a per-round basis. This result further demonstrates the energy efficiency of the robust Gur game.

In summary, our analysis of the simulation results reported in Section 4 and this section highlights the following: (1) the proposed robust Gur game demonstrates excellent reliability and QoS performance; (2) it is significantly more robust under network dynamics, such as mobility and node failure, compared to the original Gur game and shuffle; and (3) it achieves substantially higher energy efficiency than both Gur game and shuffle. Overall, the proposed robust Gur game significantly enhances QoS control and energy efficiency in WSNs, outperforming the original Gur game and shuffle.

## 6. Conclusions and Future Work

The standard Gur game is a mathematical model designed for self-organization and self-optimization, assuming that players are not constrained by resource limitations. When the Gur game is applied to WSNs for QoS control, the nature of resource-limited players (i.e., sensor nodes) raises a great challenge. In this paper, we present novel extensions and modifications of the standard Gur game and, hence, develop an improved model called the robust Gur game to address the resource issues of the original Gur game. The significant new features of our proposed robust Gur game are player rotation, a proactive referee, and unambiguous award/punishment mechanisms. In particular, the extension of unambiguous award/punishment theoretically guarantees the convergence of the robust Gur game, and, thus, has effectively addressed existing open M/2 problem of the standard Gur game. We study the behaviors of our robust Gur game by TinyOS-based WSN simulations to achieve the high fidelity of performance evaluation. Our rigorous simulation results highlight the significant potential of the proposed robust Gur game-based scheme for energy-efficient QoS control in WSNs. The robust Gur game enables sensor nodes to operate at substantially lower energy consumption rates, resulting in much higher energy efficiency. Additionally, it delivers significantly improved QoS performance and robustness on dynamic network conditions compared to the original Gur game and shuffle. Furthermore, the simulations demonstrate the robust Gur game’s unique QoS robustness, a highly desirable feature for real-world WSN applications.

A potential limitation of our method is the presence of additional parameters, such as timers, which need to be configured, as outlined in Table 5. Based on our findings, configuration 2 in Table 5 appears to be a favorable choice. While these additional parameters offer greater flexibility and design space for optimization, they also introduce a slight increase in complexity. In future work, we plan to explore the robust Gur game in multi-hop WSNs, including mesh networks and cluster topologies.

## Figures and Tables

**Table 1:** Simulation configurations.

	TOSSIM	Avrora
Network size	100	100
Optimal fraction	0.35	0.35
Round interval	15 s	15 s
Simulation time	2000 rounds	10,000 s

**Table 2:** Gur game (N = 1) QoS simulation statistics.

Performance Metrics	Trials					Avg.
	1	2	3	4	5	
QoS ratio (%)	92.90	72.35	90.15	64.65	82.55	80.52
Active nodes (avg.)	35.77	38.12	36.05	39.22	37.25	37.28
Convergence time (rounds)	163	362	129	543	13	242

**Table 3:** Gur game (N = 3) QoS simulation statistics.

Performance Metrics	Trials					Avg.
	1	2	3	4	5	
QoS ratio (%)	99.80	99.85	99.85	0.0	0.03	59.91
Active nodes (avg.)	36.96	33.96	33.97	30.59	38.96	34.89
Convergence time (rounds)	4	3	3	3	4	3.4

**Table 4:** Shuffle (N = 2) QoS simulation statistics.

Performance Metrics	Trials					Avg.
	1	2	3	4	5	
QoS ratio (%)	49.65	59.15	45.65	44.00	18.35	43.46
Active nodes (avg.)	40.70	39.58	39.45	39.86	42.16	40.35

**Table 5:** Robust Gur game time-out parameter configurations (in rounds).

Config.	Active Timer	Standby Timer	A-Sleep Timer	S-Sleep Timer
1	60	60	30	30
2	60	60	[1, 60]	[1, 60]
3	60	60	[1, 30]	[1, 30]
4	30	60	[1, 60]	[1, 60]
5	30	60	[1, 30]	[1, 30]

**Table 6:** Robust Gur game QoS ratio (%) for different time-out parameter configurations.

Config.	trials					Avg. QoS Ratio	Avg. Awake Nodes
	1	2	3	4	5		
1	71.35	68.9	73.55	70.3	78.05	72.43	60.95
2	93.70	91.95	91.90	93.60	92.10	92.65	69.36
3	85.95	86.60	84.75	85.80	83.85	85.39	81.98
4	56.80	54.05	54.65	53.95	56.55	55.30	63.77
5	78.50	76.10	77.30	76.80	74.55	76.65	77.31

**Table 7:** Robust Gur game QoS simulation statistics, configuration 2.

Performance Metrics	Trials					Avg.
	1	2	3	4	5	
QoS ratio (%)	93.7	91.95	91.9	93.6	92.1	92.65
Active nodes (avg.)	34.66	34.61	34.62	34.79	34.76	34.69
Convergence time (rounds)	5	5	4	4	5	4.6

**Table 8:** Gur game energy consumption (J) statistics for 100 nodes, 10,000 s.

N	Max	Min	Std. Dev.	Avg.
N = 1	686.12	685.84	0.063	686.00
N = 3	686.19	685.85	0.077	686.05

**Table 9:** Gur game (N = 3) energy consumption (J) of the most active node and the least active node.

Node	CPU	Radio		Others	Total
		Receive	Transmit		
Most active	101.07	563.59	0.32	21.06	686.04
Least active	100.85	563.94	0.0	21.06	685.85

**Table 10:** Robust Gur game energy consumption (J) statistics.

Trials	Max	Min	Std. Dev.	Avg.	Avg. Awake Nodes Per Round
1	634.27	389.21	50.52	509.49	71.05
2	575.40	432.42	34.49	495.79	70.93
3	588.92	407.63	34.85	490.19	70.00
Trials avg.	599.53	409.75	39.95	498.49	70.66

## Data Availability

Data is contained within the article.

## Appendix A

**Proof of the Lemma** **1.** Let reward probability p(f) f, while 0≤f≤1, 0≤p(f)≤1, and $p\left(f^{*}\right)=1$. We will prove that for $f^{*}<\frac{1}{2}$ (For $f^{*}>\frac{1}{2}$, it can be proved in a similar way),

(A1) $$\underset{T\to \infty }{\lim}\left|f\left(T\right)-f^{*}\right|=0$$

where T represents the number of epochs.

Since p(f) is a convex function, we always have the following:

(A2) $$\left\{\begin{array}{c} p\left(f\right)\geq \frac{f}{f^{*}},\;\;\;\;\;\;\;\;\;\;\;\;\;\;\;\;\;0\leq f<f^{*}\\ p\left(f\right)\geq \frac{1-f}{1-f^{*}},\;\;\;\;\;\;\;\;\;\;f^{*}<f\leq 1 \end{array}\right.$$

Now we prove that $∆f=|f\left(t\right)-f^{*}|$ will decrease as T increases, thus the system will converge to $f^{*}$.

(a) If at epoch t we have $f\left(t\right)>f^{*}$, then let $f\left(t\right)=f^{*}+∆f$. In this case, only the nodes who voted yes in the robust Gur game will change their state, s.t.

$$f\left(t+1\right)=f(t)\cdot p(f\left(t\right))<f(t)$$

If $f\left(t+1\right)>f^{*}$, we have $\left|f\left(t+1\right)-f^{*}\right|=f\left(t+1\right)-f^{*}<f\left(t\right)-f^{*}$.

If $f\left(t+1\right)<f^{*}$, we have $f(t)\cdot p(f\left(t\right))<f^{*}$, and combine with (A2), we get:

$$\frac{1-f(t)}{1-f^{*}}\leq p(f\left(t\right))<\frac{f^{*}}{f(t)}$$

$$\left(1-f\left(t\right)\right)f(t)<(1-f^{*})f^{*}$$

$$\frac{1}{4}-{\left(f(t)-\frac{1}{2}\right)}^{2}<\frac{1}{4}-{\left(f^{*}-\frac{1}{2}\right)}^{2}$$

Since $f^{*}<\frac{1}{2}$, then $\left|f(t)-\frac{1}{2}\right|>\frac{1}{2}-f^{*}$.

If $f\left(t\right)<\frac{1}{2}$, then we have $f\left(t\right)<f^{*}$, which is contradictory to $f\left(t\right)>f^{*}$. So $f\left(t\right)\geq \frac{1}{2}$, then $f(t)-\frac{1}{2}>\frac{1}{2}-f^{*}$, therefore, $f\left(t\right)>1-f^{*}$.

Now we know only if $f\left(t\right)>1-f^{*}$, then $f\left(t+1\right)$ could be smaller than $f^{*}$.

Next, we will prove that when $f\left(t+1\right)<f^{*}<f(t)$, we can have $f\left(t\right)-f\left(t+1\right)<2\cdot ∆f$.

$$\begin{array}{cc} f\left(t\right)-f\left(t+1\right) & =f\left(t\right)-f\left(t\right)\cdot p\left(f\left(t\right)\right)\\ & =f(t)(1-p\left(f\left(t\right)\right)\\ & <f\left(t\right)\left(1-\frac{1-f\left(t\right)}{1-f^{*}}\right)\\ & =f\left(t\right)\frac{f\left(t\right)-f^{*}}{1-f^{*}}=\frac{f\left(t\right)}{1-f^{*}}∆f\\ & <\frac{1}{1-\frac{1}{2}}∆f=2\cdot ∆f, \end{array}$$

s.t. $f\left(t\right)-f\left(t+1\right)<2\cdot ∆f=2(f(t)-f^{*})$. Then $0<f^{*}-f\left(t+1\right)<f(t)-f^{*}$.

Now, we have proved that if $f\left(t\right)>f^{*}$, then we always have $\left|f\left(t+1\right)-f^{*}\right|<f\left(t\right)-f^{*}$.

(b) If at epoch t we have $f\left(t\right)<f^{*}$, then let $f\left(t\right)=f^{*}-∆f$. As we know, in this case only the node who voted no will change their state, s.t.

$$f\left(t+1\right)=f\left(t\right)+(1-f\left(t\right))\cdot (1-p\left(f\left(t\right)\right))>f(t)$$

If $f\left(t+1\right)<f^{*}$, then $\left|f\left(t+1\right)-f^{*}\right|=f^{*}-f\left(t+1\right)<f^{*}-f\left(t\right)$.

If $f\left(t+1\right)>f^{*}$, it is possible that $f\left(t+1\right)-f^{*}\geq f^{*}-f\left(t\right)$, and, in this case, similar to *(a)* we should have $f\left(t+1\right)-f\left(t\right)\geq 2\cdot ∆f$. From (5), we can obtain:

$$\begin{array}{ll} 2\cdot ∆f\leq f\left(t+1\right)-f\left(t\right) & =\left(1-f\left(t\right)\right)\cdot \left(1-p\left(f\left(t\right)\right)\right)\\ & <\left(1-f\left(t\right)\right)\cdot (1-\frac{f\left(t\right)}{f^{*}})\\ & =\left(1-f\left(t\right)\right)\cdot \frac{f^{*}-f}{f^{*}}\\ & =\frac{\left(1-f\left(t\right)\right)}{f^{*}}\cdot ∆f \end{array}$$

So, $1-f\left(t\right)>2f^{*}>2f\left(t\right)$, therefore, we obtain $f\left(t\right)<\frac{1}{3}$.

Now we know only if $f\left(t\right)<\frac{1}{3}$, it is possible that $f\left(t+1\right)-f^{*}\geq f^{*}-f\left(t\right)$.

That is, once $f\left(t\right)\geq \frac{1}{3}$, we always have $f\left(t+1\right)-f^{*}<f^{*}-f\left(t\right)$.

(c) From *(a)* and *(b)*, we learn that if $f\left(t\right)\geq \frac{1}{3}$, when T increases, $\left|f\left(T\right)-f^{*}\right|$ always decreases. Now we consider the case when $f\left(t\right)<\frac{1}{3}$, we know now $f\left(t\right)<f^{*}<f\left(t+1\right)$.

From (a), we know once if $f\left(t+1\right)\leq 1-f^{*}$, for any T>t+2, $f\left(T\right)\geq f^{*}$. If $f\left(t+1\right)>1-f^{*}$, and $f\left(t+2\right)<f^{*}$, we can prove that $f\left(t\right)<f(t+2)$.

First we show that when $f\left(t\right)<f^{*}<\frac{1}{2},$ we have $f\left(t\right)+f\left(t+1\right)<1$.

$$\begin{array}{ll} f\left(t\right)+f\left(t+1\right) & =2f\left(t\right)+\left(1-f\left(t\right)\right)\cdot \left(1-p\left(f\left(t\right)\right)\right)\\ & <2f\left(t\right)+\left(1-f\left(t\right)\right)\cdot \left(1-\frac{f\left(t\right)}{f^{*}}\right)\\ & =\frac{f^{*}-f\left(t\right)\left(1-f\left(t\right)-f^{*}\right)}{f^{*}}\\ & <\frac{f^{*}}{f^{*}}=1; \end{array}\;\begin{array}{ll} f\left(t+2\right) & =f\left(t+1\right)\cdot p\left(f\left(t+1\right)\right)\\ & >f\left(t+1\right)\cdot \frac{1-f\left(t+1\right)}{1-f^{*}}\\ & >\frac{f\left(t+1\right)}{1-f^{*}}\cdot f\left(t\right)>f(t). \end{array}$$

So, if $f\left(t\right)<\frac{1}{3}$, then there exists k, when T>t+2k, we have either $f\left(T\right)\geq f^{*}$ or $f\left(T\right)\geq \frac{1}{3}$.

From *(a)*, *(b),* and *(c)*, we have $\underset{T\to \infty }{\lim}\left|f\left(T\right)-f^{*}\right|=0$. □
